# The Effects of Postmenopausal Hormone Use on Cataract: A Meta-Analysis

**DOI:** 10.1371/journal.pone.0078647

**Published:** 2013-10-24

**Authors:** Kairan Lai, Jiantao Cui, Shuang Ni, Yidong Zhang, Jiliang He, Ke Yao

**Affiliations:** 1 Eye Center, Second Affiliated Hospital, School of Medicine, Zhejiang University, Hangzhou, China; 2 Institutes of Environmental Medicine, School of Medicine, Zhejiang University, Hangzhou, China; 3 Zhejiang Provincial Key Laboratory of Ophthalmology, Hangzhou, China; Iran University of Medical Sciences, Iran (Islamic Republic Of)

## Abstract

**Background:**

Cataract is the leading cause of blindness worldwide. Many observational studies assessed the relationship between postmenopausal hormone replacement therapy (HRT) and risk of cataract development, but the reported results were controversial. The aim of present meta-analysis was to evaluate the association of postmenopausal hormone replacement therapy with risk of cataract development.

**Methods:**

The eligible observational studies, including cross-sectional, case–control and cohort studies, were identified by searching PubMed and Embase during March of 2013. Either a fixed- or a random-effects model was used to calculate the pooled odds ratio (OR) with its 95% confidence interval (95%CI). Subgroup analysis on cataract types was performed.

**Results:**

A total of four cohort and five case-control or cross-sectional studies were finally included into this meta-analysis. Overall, a significant decreased risk of developing any type of cataract was found in ever HRT group as compared with non-HRT group among cohort studies (OR 0.83; 95%CI: 0.71,0.97) and case-control or cross-sectional studies (OR 0.74; 95%CI: 0.59,0.93). Subgroup analysis on cataract types determined that the significantly decreased risk of nuclear cataract in current HRT group (OR 0.72; 95%CI: 0.61,0.85) and also a critically reduced risk of nuclear cataract in ever HRT group (OR 0.80; 95% CI: 0.64,1.01) were found among case-control or cross-sectional studies, as compared with non-HRT group. No association of HRT with risk of cortical and posterior subcapsular cataract was observed.

**Conclusions:**

The results of present meta-analysis indicate that postmenopausal hormone use may play a protective role in cataract development.

## Introduction

Cataract is the major cause of visual impairment and blindness in older adults in the world [1]. As the world’s population is aging, the prevalence of cataract is also increasing. The cataract is a significant global problem and challenge. Data from Australia and the Barbados eye studies indicated that female gender is one of the risk factors for cortical and nuclear cataract [2,3]. A number of previous epidemiologic studies have also shown an increased prevalence of cataract in women compared with men [4–7]. It was suggested that the estrogen may play a role in the cataract formation and progression.

Hormone replacement therapy is widely used in the treatment of menopausal symptoms [8], although recent studies have shown the consistent evidence of an increased risk for breast cancer and endometrial cancer in females with long-term use of hormone replacement therapy [9–11]. Evidence from laboratory studies has suggested that estrogen may protect against the development of cataract [12,13]. However, the results from epidemiological studies indicated that the association of HRT with risk of cataract was inconclusive. Some studies have demonstrated that HRT was associated with a decreased prevalence of lens opacities [14–18]. On the contrary, several studies reported no protective effect of HRT on cataract development [19–21]. The individual studies may be restricted in terms of the sample size. Therefore, in present investigation the methods of meta-analysis of the published observational studies were utilized to analyze the relationship between HRT and the prevalence of cataract, in order to provide high-quality evidence for potential therapeutic options.

## Materials and Methods

### 1: Search strategy and Selection of the Papers

The meta-analysis was performed, according to the PRISMA in systematic review and meta-analysis [22]. PubMed and Embase have been searched for original papers concerning the effects of postmenopausal hormone use on cataract until March 10, 2013. The search strategy was composed of cataract (e.g. cataract, lens opacity and crystalline opacity), hormone replacement (e.g. hormone replacement therapy, estrogen replacement therapy and estrogens) and human studies. Furthermore, the reference lists of the selected relevant papers were screened by hand for potentially relevant new papers. Cross-sectional, case–control or cohort studies incorporated in the present meta-analysis should meet the following inclusion criteria: (1) original papers which reported independent data; (2) the studies which considered the postmenopausal females as an independent study population for analysis; (3) the studies which estimated the effects of HRT on the risk of cataract with odds ratio (OR) or relative ratio (RR) and its 95% confidence interval (CI). Papers were excluded on the basis of following criteria: (1) Non-original paper (e.g. review, letter or comment etc.); (2) Non-human investigation (e.g. animal study or vitro study); (3) Anti-estrogen medication (e.g. tamoxifen) for treatment of other diseases; (4) Double publication. In case the published papers used the same database to assess the relationship between exposure and outcome, only the most recent or informative one was included.

### 2: Data Extraction and Quality Assessment

The following data were extracted independently by two authors (K. Lai and J. Cui) from each study : first author’s name, year of publication, country, study period, study design, sample size, age, cataract definitions and grading, HRT status, adjusted variables, and OR/RR values with 95% CI. The results were compared, and conflicting evaluations were discussed among all authors and resolved with consensus. HRT status was divided into current HRT, past HRT and ever HRT. Current HRT had a history of HRT with HRT prescription used currently. Past HRT had a history of HRT with no current HRT prescription. Ever HRT had a history of HRT regardless of current HRT use. As the outcome of interest, cataract is a disease presented with opacity in the lens or capsule of the eye, which leads to a decreased vision. Cataract mainly includes three subtypes, i.e. cortical cataract, nuclear cataract and post subcapsular cataract (PSC). The patients were diagnosed through examination at a slit lamp by ophthalmologists according to grading systems. 

Our primary analysis compared the risk of cataract between ever HRT users and never HRT users. Several studies did not provide an overall OR/RR for ever HRT users, but showed the separate adjusted odds ratio of different duration of postmenopausal hormone use or HRT status (past and current). And some studies did not report an OR/RR/HR for any type of cataract, but for subtypes (cortical cataract, nuclear cataract and posterior subcapsular cataract). For reason given above, Jan Hamling’s method [23] was used to estimate the adjusted overall OR/RR on the basis of Greenland and Longnecker’s effective numbers approach. In addition, the relationship between HRT status and the subtypes of cataract was estimated. Quality assessment for the included studies in this meta-analysis was performed using the Newcastle Ottawa scale (NOS) [24]. The studies that met 5 or more of the NOS criteria were considered as high quality.

### 3: Statistical Methods

OR with its 95%CI was used as a common measure for the association of HRT with risk of cataract across studies. The RR was directly considered as OR. Cochran's Q-statistic and I^2^ score [25] were utilized to assess possible heterogeneity among the individual studies. When the P-value for heterogeneity is < 0.10 or I^2^ is > 50%, substantial heterogeneity was detected. The fixed-effects model (the inverse variance method [26]) was used when no heterogeneity was observed throughout included studies. Otherwise, the random-effects model (DerSimonian and Laird method [27]) was used. 

Subgroup analysis was conducted regarding the association of HRT status (past and current use) with risk of cataract subtypes (cortical cataract, nuclear cataract and posterior subcapsular cataract), respectively. 

Potential publication bias was assessed by the Egger’s linear regression test [28] and the Begg’s rank correlation test [29]. The statistical software was Stata version 11.0 (Stata Corporation, College Station, TX), and the significance level was set to *P* < 0.05 or *P*<0.01.

## Results

### 1: Characteristics of studies

**Figure 1:**
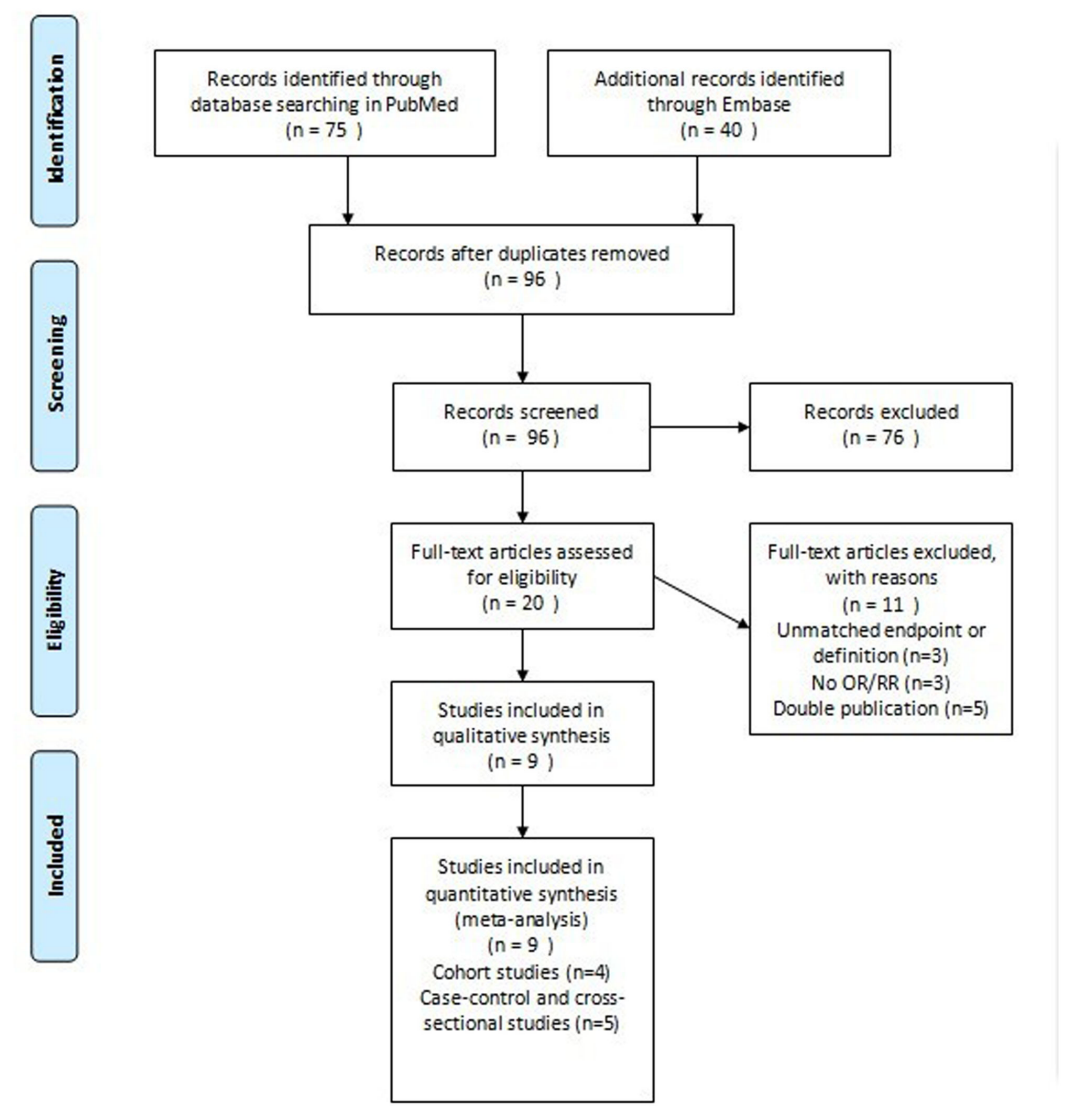
Flow diagram of literature search in this meta-analysis.

The search strategy retrieved 96 unique citations from MEDLINE (PubMed) and EMBASE databases. Of these, 76 were excluded after reviewing titles and abstracts, 20 articles for full-text review were left. In this review, 11 articles were excluded for following reasons: one article was a lens transmittance study [30], one article provided Hazard ratio (HR) instead of OR/RR [31], two articles did not provide OR and its 95% CI or sufficient information to estimate a summary OR and its 95% CI [21,32], one article identified cataract subtypes as water clefts and retrodots [33], in one article the cataract extraction served as the measure of outcome [34], and the data used in five articles were the same as other studies [15,17,35–37]. Finally, four cohort studies [18,38–40], two case-control studies [14,41] and three cross-sectional studies [3,16,19] were included in our meta-analysis. Figure 1 shows a flow of search results.

The characteristics of the included studies were presented in table 1. Among the 9 included studies, four, two, two and one studies were conducted in USA, Europe, Australia and Asia, respectively. Seven studies were the population-based studies, one was the hospital-based study and one was a selected group-based study. Different standardized criteria were used for diagnosis of cataract in some studies, while the cases in other studies were diagnosed medically by ophthalmologist or medical record review. According to NOS system, 9 included studies were considered as high quality.

**Table 1:** Characteristics of 9 studies included into this meta-analysis.

**Reference**	**Country**	**Study design**	**Sample size (females)**	**Age**	**Cataract types**	**Case criteria**	**HRT Status**	**Adjusted variables**
Klein 2000	USA	population-based cohort	1132	65 (mean)	Any type	Wisconsin Cataract Grading System	Duration of HRT	Age
Worzala 2001	USA	population-based cohort	529	66-93	Any type, nuclear, cortical, PSC	Standardized Grading System by Taylor and West	Duration of estrogen use	Age, type of menopause, age at menarche, diabetes, BMI, hypertension and cigarette smoking
Weintraub 2002	USA	selected group-based cohort	480	63 (mean)	Any type, nuclear, cortical	LOCS III	Ever, past, current	Age, pack years of smoking, average vitamin C intake and BMI
Kanthan 2010	Australia	population-based cohort	1159	59+	Nuclear, cortical, PSC	Wisconsin Cataract Grading System	Past, Current	Age, smoking, use of oral or inhaled steroids, socioeconomic status, myopia, hypertension and diabetes.
Aina 2006	UK	population-based case-control	20000	81 (mean)	Any type	None shown	Past, Current	Age, consultation rate
Noran 2007	Malaysia	hospital-based case-control	242	63.8 (mean)	Any type	None shown	Duration of estrogen use	Age, ethnic, education, smoking status, alcohol consumption, family history of cataract, aspirin and steroids intake, diabetes mellitus, hypertension, and other reproductive factors
McCarty 1999	Australia	population-based cross-sectional	2850	61 (mean)	Nuclear, cortical	Wilmer Cataract Grading System	Ever	Age, education occupation, hypertension, diabetes, BMI, arthritis, smoking history, alcohol use, et al.
Freeman 2001	USA	population-based cross-sectional	1239	65-84	Any type Nuclear, cortical, PSC	Wilmer Cataract Grading System	Past, Current	Age, race, hypertension, smoking, alcohol consumption, age at menopause, age at menarche, diabetes, steroid use, hysterectomy, BMI, education, number of births, use of birth control pills, et al.
Defay 2003	France	population-based cross-sectional	1410	60-93	Any type, nuclear, cortical, PSC	LOCS III	Past, Current	Age, education, brown iris, smoking, diabetes mellitus, corticosteroid therapy, asthma or chronic bronchitis, plasma retinol, plasma glutathione peroxidase, erythrocyte superoxide dismutase activity and sunlight exposure

HRT: hormone replacement therapy; PSC: posterior capsular cataract; LOCS III: Lens Opacities Classification System III; BMI: body mass index.

### 2: Cohort studies

**Figure 2:**
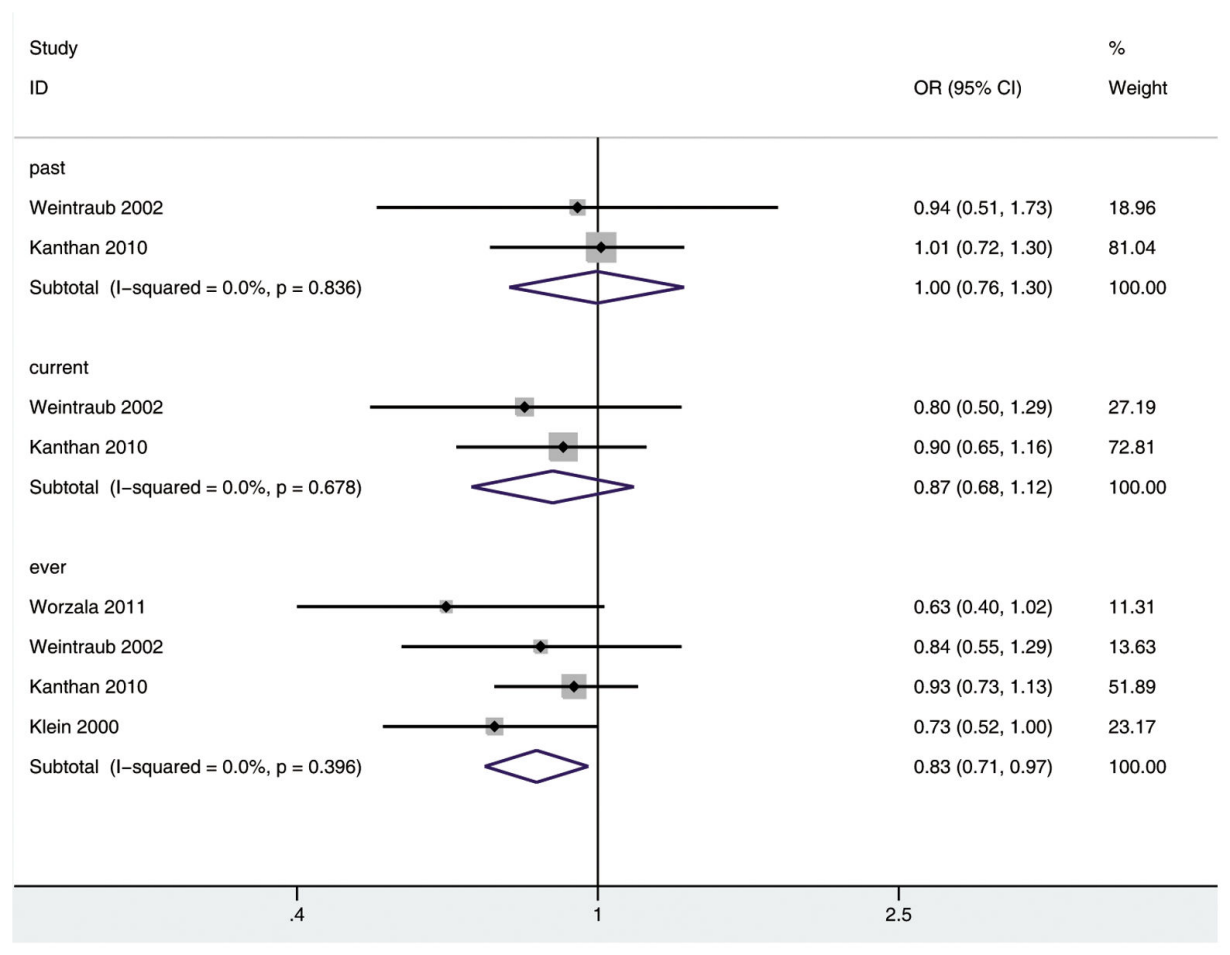
The association of HRT with risk of any type of cataract in cohort studies.

In four [18,38–40], two [38,40] and two [38,40] studies the analysis of ever, past, current postmenopausal hormone use and risk of any type of cataract was performed, respectively. Figure 2 shows there was a statistically significant decrease for the association of ever HRT with risk of any type of cataract in a fixed-effects model (OR 0.83; 95% CI: 0.71, 0.97; *P*<0.05). But there was no significant difference for developing any type of cataract in past HRT group (OR 1.00; 95% CI: 0.76, 1.30), current HRT group (OR 0.87; 95% CI: 0.68, 1.12), as compared with non-HRT group. There was no substantial heterogeneity among the included studies (ever HRT group: *P*=0.396, I^2^=0.0%; past HRT group: *P*=0.836, I^2^=0.0%; current HRT group: *P*=0.678, I^2^=0.0%). No publication bias was found among the four included studies (Begg, *P*=0.308; Egger, *P*=0.188).

Three [18,38,40], two [38,40] and two [38,40] studies were included in the analysis of the risk of nuclear cataract in past, current and ever HRT groups, respectively. There was no significant difference for developing nuclear cataract in past HRT group (OR 1.20; 95% CI: 0.87, 1.67), current HRT group (OR 0.80; 95% CI: 0.59, 1.07) and ever HRT group (OR 0.86, 95% CI: 0.69, 1.07), as compared with non-HRT group. There was no substantial heterogeneity among the included studies (ever HRT group: *P*=0.233, I^2^=31.4%; past HRT group: *P*=0.204, I^2^=38.0%; current HRT group: *P*=0.804, I^2^=0.0%). Publication bias was found among the four included studies by the Egger’s linear regression test (*P*=0.003), but not by the Begg’s rank correlation test (*P*=0.296).

Three [18,38,40], two [38,40] and two [38,40] studies were included in the analysis of the risk of cortical cataract in past, current and ever HRT groups, respectively. There was no significant difference for developing cortical cataract in past HRT group (OR 0.91; 95% CI: 0.67, 1.23), current HRT group (OR 0.90; 95% CI: 0.68, 1.19) and ever HRT group (OR 0.92; 95% CI: 0.75, 1.13), as compared with non-HRT group. There was no substantial heterogeneity among the included studies (ever HRT group: *P*=0.539, I^2^=0.0%; past HRT group: *P*=0.319, I^2^=0.0%; current HRT group: *P*=0.412, I^2^=0.0%). No publication bias was found among this subgroup (Begg, *P*=1.000; Egger, *P*=0.993).

Two [18,40] studies were included in the analysis of the association between risk of post subcapsular cataract and ever HRT. Substantial heterogeneity was observed (*P*=0.090, I^2^=65.2%). The summary OR was 0.67 (95% CI: 0.35, 1.29) with a random-effects model.

### 3: Case-control and cross-sectional studies

**Figure 3:**
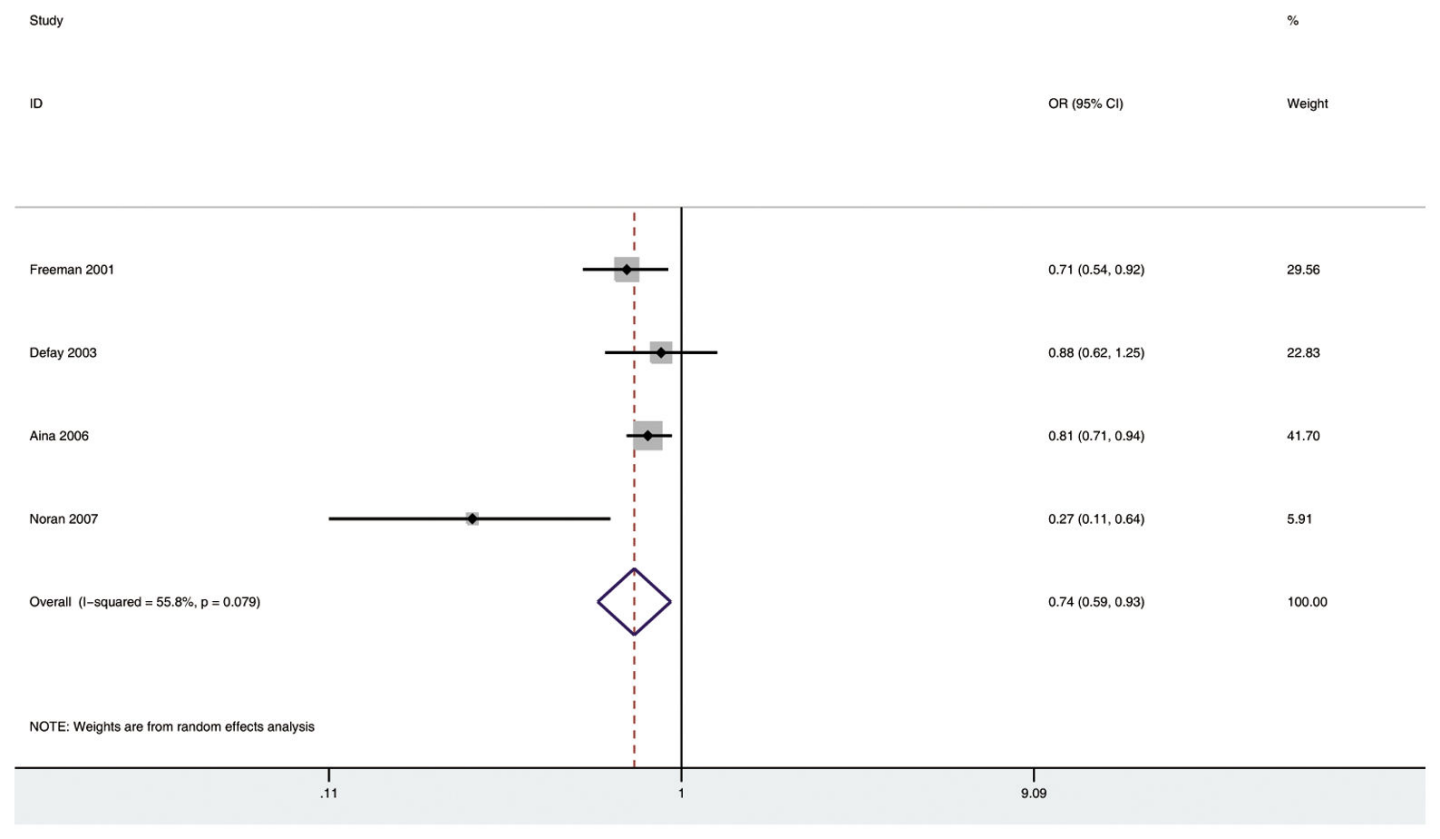
The association of ever HRT with risk of any type of cataract in case-control and cross-sectional studies.

Four [14,16,19,41] studies were included in the analysis of ever postmenopausal hormone use and risk of any type of cataract. Figure 3 shows a statistically significant decrease for the association of ever HRT with risk of any type of cataract in a random-effects model (OR 0.74; 95% CI: 0.59, 0.93; *P*<0.05). Substantial heterogeneity was observed (*P*=0.079, I^2^=55.8%). Further scrutiny indicated that the heterogeneity shifted from *P*=0.079 to *P*=0.582 by Q test and I^2^ score shifted from 55.8% to 0.0% when the only study conducted in Asia with a small sample size (Noran, et al.) was excluded (OR 0.80; 95% CI: 0.71,0.90; *P*<0.01; a fixed-effects model). No significant publication bias was found among the four included studies (Begg, *P*=0.308; Egger, *P*=0.267).

**Figure 4:**
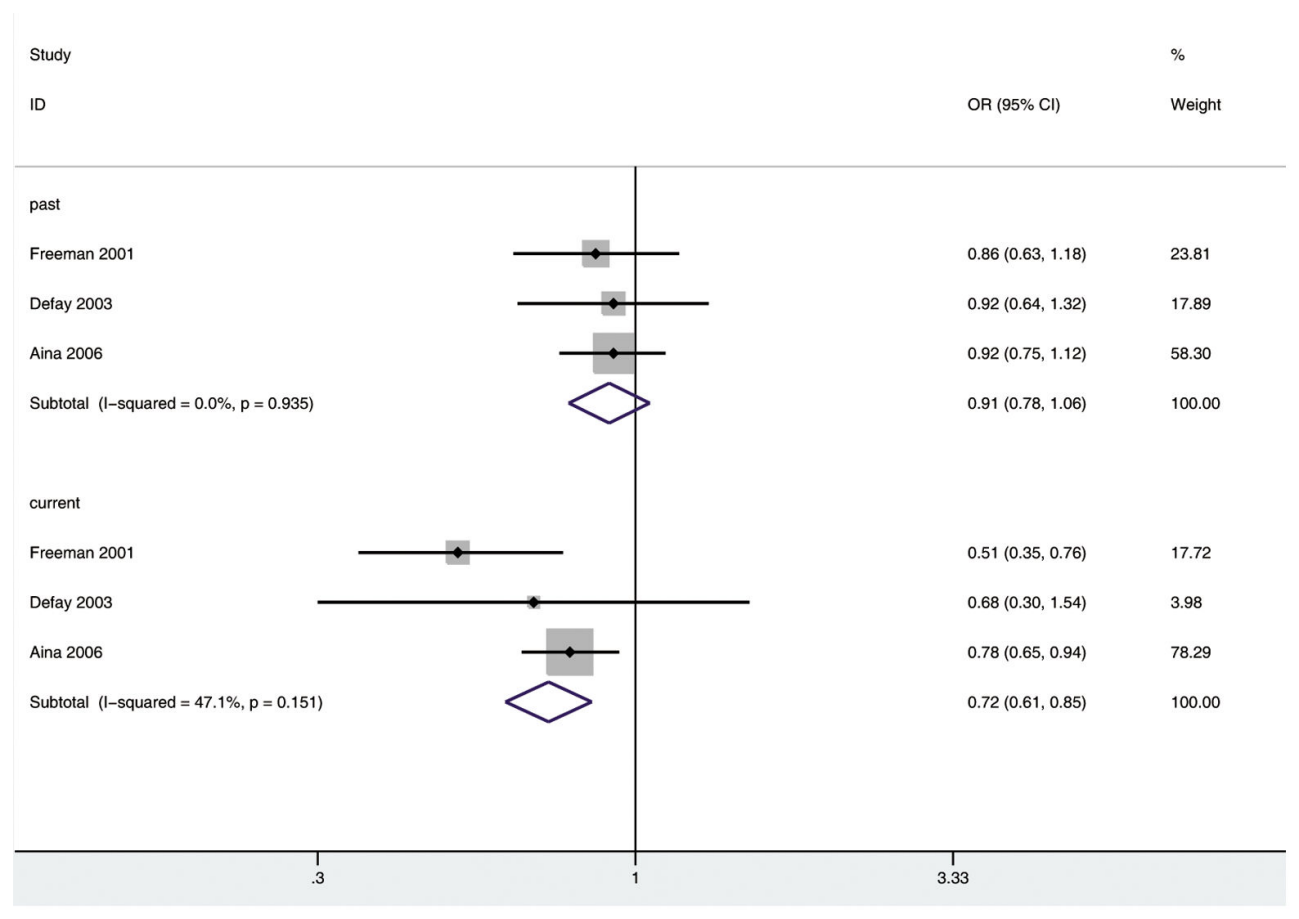
The association of past or current HRT with risk of any type of cataract in case-control and cross-sectional studies..

Three [14,16,19] studies were included in the separate analysis of the association of past or current HRT with risk of any type of cataract. There was a significantly decreased risk of developing any type of cataract in current HRT group (OR 0.72, 95% CI: 0.61, 0.85; *P*<0.01), but not in past HRT group (OR 0.91, 95% CI: 0.78, 1.06), as compared with non-HRT group (Figure 4). There was no substantial heterogeneity among the included studies (past HRT group: *P*=0.968, I^2^=0% and current HRT group: *P*=0.117, I^2^=43.3%).

**Figure 5:**
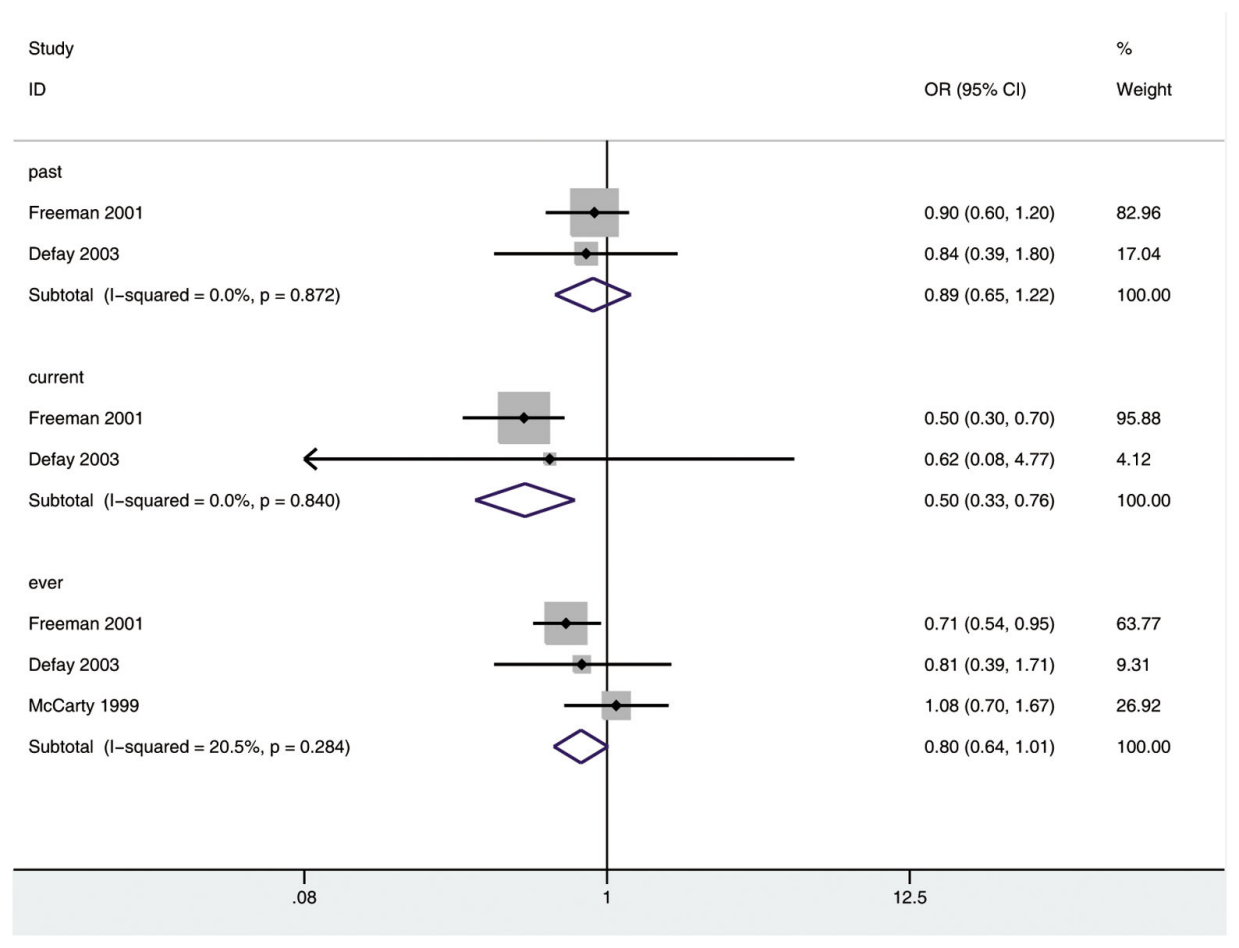
The association of HRT with risk of nuclear cataract in case-control and cross-sectional studies.

Three [3,16,19], two [16,19] and two [16,19] studies were included in the analysis of the risk of nuclear cataract in past, current and ever HRT groups, respectively. There was a significantly decreased risk of developing nuclear cataract in current HRT group (OR 0.50; 95% CI: 0.33, 0.76; *P*<0.01) and a critically decreased risk in ever HRT group (OR 0.80; 95% CI: 0.64, 1.01; *P*=0.059), but not in past HRT group (OR 0.89; 95% CI: 0.65, 1.22), as compared with non-HRT group (Figure 5). There was no substantial heterogeneity among the included studies (past HRT group: *P*=0.872, I^2^=0%; current HRT group: *P*=0.840, I^2^=0%; past HRT group: *P*=0.284, I^2^=20.5%). No significant publication bias was found among the three included studies (Begg, *P*=1.000; Egger, *P*=0.670).

The studies included in the analysis of the risk of cortical cataract were the same as those in the analysis of nuclear cataract for each group. There was no significant difference for developing cortical cataract in past HRT group (OR 1.02; 95% CI: 0.71, 1.47), current HRT group (OR 0.67; 95% CI: 0.37, 1.22) and ever HRT group (OR 0.93; 95% CI: 0.72, 1.18), as compared with non-HRT group. No substantial heterogeneity was detected among the included studies (ever HRT group: *P*=0.429, I^2^=0.0%; past HRT group: *P*=0.666, I^2^=0.0%; current HRT group: *P*=0.783, I^2^=0.0%). No publication bias was found among this subgroup (Begg, *P*=1.000; Egger, *P*=0.054).

## Discussion

The results of present meta-analysis showed that postmenopausal hormone use was associated with a decreased risk of cataract without regard to the cataract type for ever HRT users in cohort and case-control or cross-sectional studies. Also the same association of current HRT with nuclear cataract was found in case-control or cross-sectional studies. However, no association of ever HRT with risk of cortical cataract and PSC was found in our meta-analysis. 

Considering the different status of HRT use, the current HRT users had a decreased risk of developing any type of cataract, nuclear cataract and cortical cataract, but neither any type of cataract nor subtypes were correlated with the past HRT. Because of the limited studies included in the subgroup analysis, the results should be interpreted with caution. 

Our findings are similar to a lens transmittance study conducted by Castillo et al. [30], in which 19 postmenopausal women with estrogen for more than 4 years (group 1), 20 postmenopausal women without estrogen (group 2), and 23 age-matched men (group 3) were studied. Lens transmittance values in the three groups were 0.905 ± 0.03, 0.839 ± 0.08, and 0.841 ± 0.08, respectively. There was a significant difference between group 1 and the other two groups and it was suggested that there was a protective effect of estrogen on the lenses of postmenopausal women. However, Uncu et al. reported a dissimilar finding that HRT did not affect lens opacity after treatment for 12 months[21]. 

Our meta-analysis indicated that current HRT users had a slightly lower risk of cataract as compared with past HRT users. Each article, which evaluated current and past HRT use separately, reported the same tendency [14,16,19,38,40]. One possible reason is that the exposure time and concentrations of estrogen in current HRT users may be higher than those in past HRT users. It was reported in Framingham study [18] that the increasing duration of postmenopausal estrogen use was strongly associated with a decreased prevalence of any type of cataract and nuclear cataract, and borderline association of the increasing duration of HRT with decreased risk of posterior subcapsular cataract was found. In Salisbury eye evaluation project [16], in the subjects with HRT for more than 3 years, the odds ratios were 0.6, 0.6 and 0.3 for nuclear, cortical and posterior subcapsular cataract, respectively. But In the subjects with HRT for less than 3 years, the odds ratios were 0.7, 1.4 and 0.4 for nuclear, cortical and posterior subcapsular cataract, respectively. A relative lower OR for a longer duration of HRT was also found in the POLA study [19] and a Malaysian study [41]. Also Malaysian study indicated that females exposed to endogenous estrogen for less than 32 years had a significantly higher risk of developing cataract, as compared with those exposed to endogenous estrogen for more than 32 years [41]. A similar but not significant tendency was found by the Blue Mountains Eye Study [40], the Beaver Dam Eye Study [39], and the Aravind Comprehensive Eye Survey [20].

The present meta-analysis showed that the association of HRT with risk of nuclear cataract was significantly stronger than the association of HRT with cortical and post subcapsular cataract. The human lens grows continuously throughout life by the addition of fiber cells, and the lens core faces a longer exposure to environmental risk factors, such as ultraviolet light and smoking, which induce the progressive oxidative damage of proteins. Moreover, it has been suggested that estrogen has an antioxidant properties [42] and preserve mitochondrial function, cell viability and ATP levels in human lens cells during oxidative stress [43]. Thus, estrogen may protect the lens from oxidative damage.

The mechanisms of the potential protective roles of estrogen in cataract formation are not fully understood. Besides the antioxidative effects, several other biological mechanisms may be involved. Firstly, estrogen may provide protection by a direct interaction with estrogen receptors (ERs). ER has been detected in ocular tissues, including human lens epithelial cells [44]. Davis et al. found that in the ER**Δ**3 transgenic mice, cortical cataracts spontaneously form in ER**Δ**3 females after puberty and progress with age [45]. Secondly, other articles have indicated that estrogen provides protection against cataract by countering the damaging effects induced by transforming growth factor β (TGF-β) [13,46]. TGF-β has been identified to be presented in the eye and is capable of inducing opacities in cultured rat lenses. Lenses from ovariectomized female rats showed the increased sensitivity to the damaging effects induced by TGF-β, and the estrogen replacement in vivo, or exposure to estrogen in vitro, could restore the resistance. The maintaining effect of estrogen on the normal function of cell membrane has also been considered to be a possible molecular mechanism [47].

Several limitations of present meta-analysis should be considered. First of all, the postmenopausal hormone replacement therapy was estimated by the self-report questionnaires in most studies, resulting in misclassification of exposure status and a existing recall and selection bias, confounding the association of HRT use with risk of cataract. And the definitions of HRT use (including the regimen, dosage and duration) were various in each study and contributed to an increase of heterogeneity. Secondly, the assessment of cataract or its subtypes varied between studies and a few studies even did not exhibit the case criteria and grading system. Thirdly, the adjusted factors in each study were different and some confounding factors which might account for the association of HRT with risk of cataract were not adjusted in some of the included studies. Although all studies were considered as high quality according to the corresponding quality assessment, the different study designs would inevitably lead to the increased inter-study heterogeneity. In addition, we did not try to contact the authors for original information which was not available in published form, and the estimates by statistical methodology might be a little different from the actual data. Because of the limited studies, the results should be interpreted with caution, and an update meta-analysis should be conducted with inclusion of newly published studies. 

Our study found that postmenopausal hormone use had a protective effect on cataract development. Further studies are needed to confirm these findings and make a better understanding of the biological mechanisms. Due to the reported effect of hormone replacement therapy on breast cancer, endometrial cancer, cardiovascular disease, osteoporosis and diseases of other systems, medical professionals should take all potential benefits and risks into account when considering HRT. 

## Supporting Information

Checklist S1 **PRISMA 2009 checklist in this meta-analysis.**
(DOC)
